# Cystoscopic Management of the Intravesical Migration of an Intrauterine Device Complicated by Bladder Stone Formation: A Video Case Report

**DOI:** 10.7759/cureus.62467

**Published:** 2024-06-16

**Authors:** Ekansh Gupta, Madhumohan Prabhudesai, Rajesh Halarnakar, Prashant Lawande, Veku Gaude

**Affiliations:** 1 Department of Urology, Goa Medical College, Bambolim, IND

**Keywords:** iud in the bladder, minimally invasive iud removal, urinary bladder stone, iud failure, migrated iud

## Abstract

Intrauterine device (IUD) migration is a rare complication of IUD placement. The current case is of a 32-year-old lady who presented with pregnancy following IUD failure. Subsequent imaging revealed intravesical migration of the IUD. A cystoscopic evaluation revealed a bladder stone (encrusted IUD), with no breach in the bladder mucosa and no evidence of a fistulous opening. The encrustation was broken down, and an intact IUD was retrieved. This video report shows the management of a migrated IUD complicated by a bladder stone using cystoscopy that not only allowed for a combined diagnostic and therapeutic approach to the extraction of the IUD but also provided information regarding the involvement of the bladder wall and confirmed no extravesical protrusion of the device.

## Introduction

An intrauterine device (IUD) is a widely used economical and effective contraceptive option. The popularity of IUD is at least in part attributed to its safety profile, good tolerability, and high contraceptive efficacy [1]. IUD placement, however, may also be associated with complications such as pain during insertion, abnormal bleeding, expulsion, or uterine perforation [2]. Migration of IUD is another rare complication associated with IUD placement and can be seen in up to 0.1% of the cases [3]. The duration between IUD placement and migration of the device remains highly variable and can even be as long as 42 years from the time of IUD placement [4]. The imprecise symptoms and a very low index of suspicion make the timely diagnosis of a migrated IUD quite tricky. Owing to the rarity of this complication, there is no current consensus on its management. There is an even greater sparsity of data describing the incidental detection of a migrated IUD due to contraceptive failure and subsequent pregnancy. The current video report describes a case of contraceptive failure following asymptomatic migration of the IUD, complicated by bladder stone formation around the displaced device.

## Case presentation

**Figure 1:**
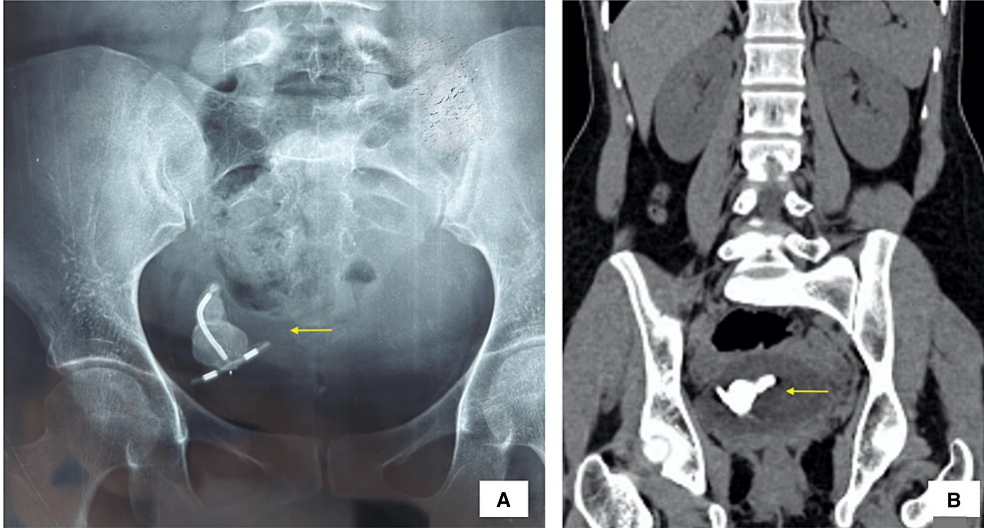
Radiological images (X-ray (A) and computed tomography scan (B)) showing the migrated IUD Arrow: Encrusted intravesical IUD IUD: intrauterine device

A 32-year-old lady presented to the Department of Obstetrics for antenatal care, following pregnancy due to contraceptive failure. She had undergone a medically induced termination of pregnancy seven years ago, following which IUD insertion was done at a local healthcare center. The post-IUD insertion period was uneventful, and the patient reported feeling the IUD threads for a few months before she stopped feeling them in the vaginal canal. Due to a lack of symptoms, she did not seek any further medical assistance. Following her delivery, she was referred to the Department of Urology in view of X-ray and sonographic findings suggestive of a displaced IUD, suspiciously located in the bladder. Subsequent computed tomography scanning revealed an intravesical calculus (~3 cm in size), with no evidence of upstream hydroureteronephrosis on either side (Figure 1).

A retrospective interaction with the patient revealed a history of mild on-and-off dysuria and hematuria, with the last episode occurring more than six months ago and resolving spontaneously. The patient did not recall any periods of urinary incontinence associated with her urological complaints.

**Figure 2:**
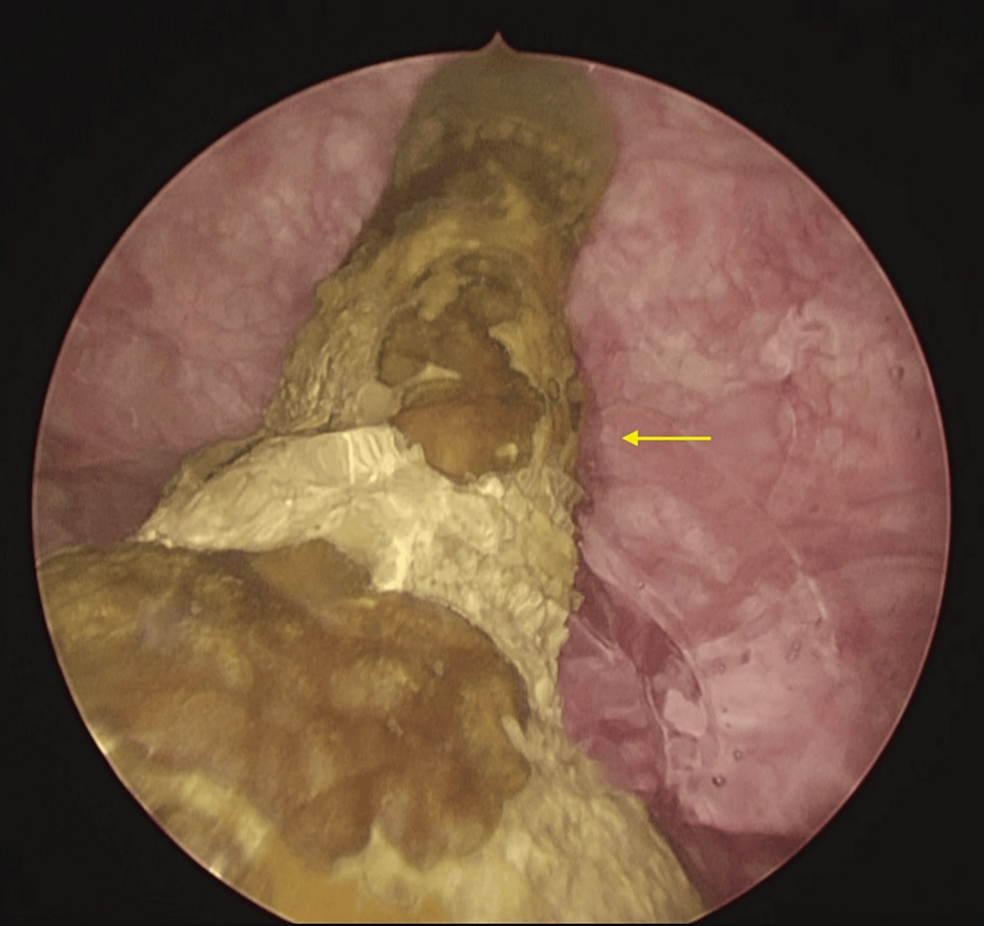
Cystoscopic view of the encrusted IUD Arrow: Encrusted intravesical IUD IUD: intrauterine device

After a thorough preoperative workup, the patient was then taken up for the cystoscopic evaluation of the bladder along with the extraction of the encrusted IUD (Figure 2, Video 1).

**Video 1:** Cystoscopic management of the intravesical migration of IUD IUD: intrauterine device

Intraoperative evaluation revealed a completely encrusted IUD (bladder stone), along with normal ureteric orifices and occasional trabeculations over the bladder wall. There was no breach in the bladder mucosa by the encrusted IUD, and no fistulous opening could be appreciated in the bladder wall. Using a pneumatic lithotripter, the stone was broken down to expose the IUD, which was then held from the tail end and retrieved through the cystoscope sheath. The patient reported no significant pain/dysuria/hematuria or bladder discomfort following the procedure and was discharged after an uncomplicated postoperative period.

## Discussion

The IUD is the most widely used reversible contraceptive, offering several advantages such as simplicity, low cost, efficacy, and safety [5]. Despite its safety, it is associated with various complications, one of which is migration into adjacent structures, although this is rare. As per the literature available so far, the incidence of IUD migration into the adjacent structures of the uterus is as low as one in 1000 insertions [6]. 

The clinical presentation of a displaced IUD remains variable, ranging from asymptomatic migration to severe lower urinary tract symptoms and, occasionally, to an unplanned pregnancy. There are two plausible explanations commonly employed to explain the intravesical migration of IUD. One is an immediate traumatic perforation, and the second is the gradual erosion of the IUD through the myometrium and into the bladder lumen, a process that may take months to years [7,8].

Transvaginal sonography is the modality of choice for identifying an intravesical IUD [9]. A contrast-enhanced computed tomography scan and magnetic resonance imaging are particularly useful tools for the diagnosis of additional complications such as a fistulous tract [10]. These walking IUDs are a gynecologist's nightmare as they pave the way for various complications. Removal of the intravesical IUD is mandatory because if left unattended, it may lead to complications such as cystitis, intravesical stone formation, pelvic abscess, and adhesions. The majority of such cases are treated by cystoscopy. Cystoscopy is particularly beneficial, as not only does it offer a dual (diagnostic and therapeutic) advantage but it also provides information regarding adherence of IUD to the bladder wall and the extent of its extravesical protrusion. However, if a part or whole of the IUD is intraperitoneal, laparoscopy/laparoscopy combined with cystoscopy/laparotomy may be chosen as the preferred therapeutic approach.

## Conclusions

While migrated intravesical IUD can be associated with lower abdominal discomfort in some cases, it may even remain asymptomatic for years before being detected following pregnancy due to contraceptive failure. The presence of a foreign body (IUD) in the bladder lumen for a prolonged duration can be complicated by bladder stone formation, which may then be treated with cystoscopic lithotripsy. Once the migrated device is adequately visualized, it is essential to remove the device in totality and ensure no breach of the bladder mucosa. Any extravesical protrusion of the device may have mandated more invasive procedures for the retrieval of the migrated IUD and repair of the breach in the bladder wall. Finally, patients should be informed of such rare complications prior to insertion of the IUD, as such cases could potentially lead to medico-legal implications later on.
